# Mitochondrial transcription factor A is a proinflammatory mediator in hemorrhagic shock

**DOI:** 10.3892/ijmm.2012.959

**Published:** 2012-04-02

**Authors:** WAYNE W. CHAUNG, RONGQIAN WU, YOUXIN JI, WEIFENG DONG, PING WANG

**Affiliations:** 1 The Feinstein Institute for Medical Research, Hofstra North Shore-LIJ School of Medicine, Manhasset, NY 11030, USA; 2 Department of Surgery, Hofstra North Shore-LIJ School of Medicine, Manhasset, NY 11030, USA

**Keywords:** mitochondrial transcription factor A, damage-associated molecular pattern, inflammation, cytokine, macrophage, hemorrhage

## Abstract

Endogenous molecules released by dying cells [i.e., damage-associated molecular patterns (DAMPs)] after trauma and severe blood loss can activate pattern recognition receptors, leading to a cascade of inflammatory responses and organ injury. Mitochondrial transcription factor A (TFAM) is a transcription factor for mitochondrial DNA. TFAM is structurally related to high mobility group box 1 (HMGB1), an important member of DAMPs. We, therefore, hypothesized that TFAM can be released into the circulation after hemorrhage to initiate inflammatory responses. In order to examine this hypothesis, male Sprague-Dawley rats were bled to and maintained at a mean arterial pressure of 40 mmHg for 90 min. They were then resuscitated with an equal volume of shed blood in the form of Ringer’s lactate (i.e., low-volume resuscitation) over 60 min. TFAM levels in the serum were measured at 4 h after hemorrhage and resuscitation. Our results showed that serum levels of TFAM were more than doubled after hemorrhage and resuscitation. To further characterize TFAM’s biological activity, we expressed recombinant rat TFAM with a GST-tag (GST-TFAM) in an *E. coli* expression system. The purity of GST-TFAM was over 99% and it was immunoreactive for specific anti-TFAM antibodies. Using RAW 264.7 cells and primary rat peritoneal macrophages, we showed that GST-TFAM dose-dependently increased TNF-α release. To determine the biological activity of GST-TFAM *in vivo*, GST-TFAM was intravenously injected in healthy male adult rats. Our results demonstrated that intravenous injection of GST-TFAM, not GST alone, upregulated circulating levels of pro-inflammatory cytokines, increased neutrophil infiltration to the lungs and caused organ injury in healthy animals. Thus, TFAM can act as a DAMP and may contribute to the initiation of inflammatory responses in hemorrhagic shock.

## Introduction

Trauma with severe blood loss represents a major clinical problem (1). Despite advances in trauma management, a large number of such patients die of severe hemorrhagic shock (2–5). Most trauma deaths result either from insufficient tissue perfusion due to excessive blood loss or the development of inflammation, infection and vital organ damage following resuscitation. Activation of the systemic proinflammatory response is an important pathophysiological component of hemorrhagic shock (6). During fluid resuscitation, excessive amount of inflammatory cytokines are produced, causing systemic inflammatory response syndrome and multiple organ dysfunction (7).

Both infection and injury can trigger a systemic inflammatory response syndrome. The response to exogenous pathogens leads to activation of innate immunity through the release of pathogen-associated molecular patterns (PAMPs) and their binding to pattern recognition receptors (8,9). On the other hand, endogenous molecules released by dying cells (damage-associated molecular patterns; DAMPs) can also activate pattern recognition receptors leading to downstream inflammation and tissue damage (8,10). In recent years, several molecules varying in both structure and intracellular function have been identified as DAMPs. Members of this growing family include high mobility group (HMG) box 1 (HMGB1) (11), heat shock proteins (12) and mitochondrial DNA (13).

Mitochondrial transcription factor A (TFAM) is a transcription factor for mitochondrial DNA. It is composed of two HMG boxes and a basic carboxyl terminal region (C-tail). Like many other HMG family proteins, TFAM has the ability to bind to DNA in a sequence-independent manner. A growing body of evidence suggests that TFAM may play a crucial role in maintaining mitochondrial DNA as a main component of the nucleoid. Although TFAM is structurally related to HMGB1, it remains unknown whether it can be identified as a DAMP. The purpose of this study, therefore, was to test the hypothesis that TFAM can be released into the circulation after hemorrhage to initiate inflammatory responses.

## Materials and methods

### Animal model of hemorrhage shock

The model of hemorrhage shock used in this experiment has been described in detail (14–16). Male Sprague-Dawley rats (275–325 g; Charles River Laboratories International, Inc., Wilmington, MA) were used in this study. The rats were housed in a temperature-controlled room on a 12-h light/dark cycle and fed on a standard Purina rat chow diet. The rats were fasted for 6 h prior to the procedure. The rats were anesthetized with isoflurane inhalation. Catheters (PE-50 tubing) were placed in the femoral vein and artery after carefully separating the femoral nerve and blood vessels. The femoral artery on the opposite side was also catheterized. One arterial catheter was used for monitoring the mean arterial pressure (MAP) and heart rate (HR) via a blood pressure analyzer (BPA; Digi-Med, Louisville, KY), the other was used for blood withdrawal and the venous catheter was used for fluid resuscitation. The rats were rapidly bled to a MAP of 40 mmHg within 10 min. The MAP was maintained for 90 min by further withdrawal of small volumes of blood or provision of small volumes of Ringer’s lactate. At the end of this hypotensive period, the rats were resuscitated with an equal volume of shed blood in the form of Ringer’s lactate (i.e., low-volume resuscitation) over a 60-min period. The shed blood was not used for resuscitation and the animals were not heparinized prior to, during and following hemorrhage. Sham-operated animals underwent the same surgical procedure but were neither bled nor resuscitated (Sham group). Animal experimentation was carried out in accordance with the Guide for the Care and Use of Laboratory Animals (Institute of Laboratory Animal Resources). This project was approved by the Institutional Animal Care and Use Committee of the Feinstein Institute for Medical Research.

### Western blot analysis of TFAM

Six microliters of serum from each sample was loaded on 4–12% Bis-Tris gels (Invitrogen Life Technologies, Carlsbad, CA) and subjected to electrophoresis using MES-SDS running buffer (Invitrogen Life Technologies). After electrophoresis, gels were transferred to 0.2 μm nitrocellulose membranes (Invitrogen Life Technologies) and blocked with 5% nonfat dry milk in 10 mM Tris-HCl with 0.1% Tween-20, pH 7.5 (TBST). The membranes were incubated with goat anti-TFAM polyclonal antibody (sc19050; Santa Cruz Biotechnology, Inc., Santa Cruz, CA) at 4°C overnight. The membranes were then washed with TBST and incubated with HRP-linked anti-goat IgG (Southern Biotech, Birmingham, AL) for 1 h at room temperature and detected using chemiluminescence and autoradiography. The intensity of the band was analyzed by the Bio-Rad GS-800 calibrated densitometer.

### Expression of recombinant rat TFAM

The coding region of rat TFAM (NM_031326) was amplified by PCR using rat cDNA clones (BC 062022) as templates. A *Bam*HI restriction site was introduced to the sense primer of TFAM (5′-CTC GGATCCATGGCGCTGTTCCGGGGAATGT-3′) to facilitate cloning. An *Xho*I restriction site was introduced to the anti-sense primer of TFAM (5′-CTCCTCGAGATTCTCAGA GATGTCTCCC-3′). The PCR product was digested with *Bam*HI and *Xho*I, then cloned into a pGEX4T3 vectors (Invitrogen Life Technologies). The sequences of TFAM with a glutathione S-transferase (GST)-tag were confirmed by DNA sequencing. The TFAM containing pGEX4T3 expression vectors were then transformed into *E. coli* competent cells BL21-Gold (Agilent Technologies, Inc., Santa Clara, CA). The GST fusion protein was purified by Glutathione sepharose 4B beads (GE Healthcare Life Sciences, Pittsburgh, PA) according to the manufacturer’s instructions. Endotoxin contamination of the protein solution was removed by phase separation using Triton X-114 (17). The content of LPS in the sample was determined using the Limulus Amebocyte Lysate assay (BioWhittaker, Inc., Walkersville, MD) as previously described (18). The purity of GST-TFAM was evaluated by SDS-PAGE on a 4–20% Tris-HCl gel and visualized using the GelCode Blue Stain Reagent (Pierce Biotechnology, Inc., Rockford, IL). The final product was concentrated by Amicon Ultra-15 Centrifugal Filter Devices to the designed concentrations and stored at −20°C.

### Cell culture

The murine macrophage cell line RAW 264.7 was purchased from the American Type Culture Collection (ATCC, Manassas, VA). Peritoneal macrophages were isolated from normal male Sprague-Dawley rats. Briefly, peritoneal macrophages were isolated by peritoneal lavage with Hanks’ balanced salt solution (HBSS) as previously described (19). RAW 264.7 cells or isolated peritoneal macrophages were cultured in DMEM containing 10% heat-inactivated fetal bovine serum, 10 mM HEPES, 100 U/ml penicillin and 100 μg/ml streptomycin at the concentration of 10^6^ cells/ml and plated at a density of 5×10^5^/well in 24-well cell culture plates. After being washed twice with HBSS, the cells were cultured in culture medium containing various concentrations of GST-TFAM (0.1, 1 and 10 μg/ml) or GST alone (5 μg/ml) for a period of 4 h. The supernatant levels of TNF-α were measured by ELISA as described below.

### Administration of GST-TFAM

To determine the effect of TFAM *in vivo*, we administered GST-TFAM to healthy rats. Briefly, male rats (275–325 g) were anesthetized with isoflurane inhalation. The femoral vein was carefully separated from the artery and cannulated with a catheter (PE-50 tubing). GST-TFAM (1 mg/kg; BW), GST (0.5 mg/kg; BW) or sham (1 ml normal saline) was administered via the femoral venous catheter over a period of 60 min. Five or 20 h later, the animals were sacrificed. Blood and tissue samples were collected.

### Determination of serum levels of TNF-α and interleukin-6 (IL-6)

Serum levels of TNF-α and IL-6 were quantified using an enzyme-linked immunosorbent assay (ELISA) kit specifically for rat TNF-α or IL-6 (BD Biosciences, San Diego, CA). The assay was carried out according to the instructions provided by the manufacturer.

### Determination of neutrophil infiltration

Neutrophil accumulation in the lung tissue was estimated using the myeloperoxidase (MPO) activity assay as previously described (20).

### Determination of serum lactate

Serum concentrations of lactate were determined using an assay kit according to the manufacturer’s instruction (Pointe Scientific, Inc., Lincoln Park, MI).

### Statistical analysis

All data are expressed as means ± SE and compared by one-way analysis of variance (ANOVA). When the ANOVA was significant, post-hoc testing of differences between groups was performed using the Student Newman-Keuls method. Student’s t-test was used when only two groups were compared. A P-value <0.05 was considered statistically significant.

## Results

### Serum levels of TFAM are increased after hemorrhagic shock in rats

**Figure 1:**
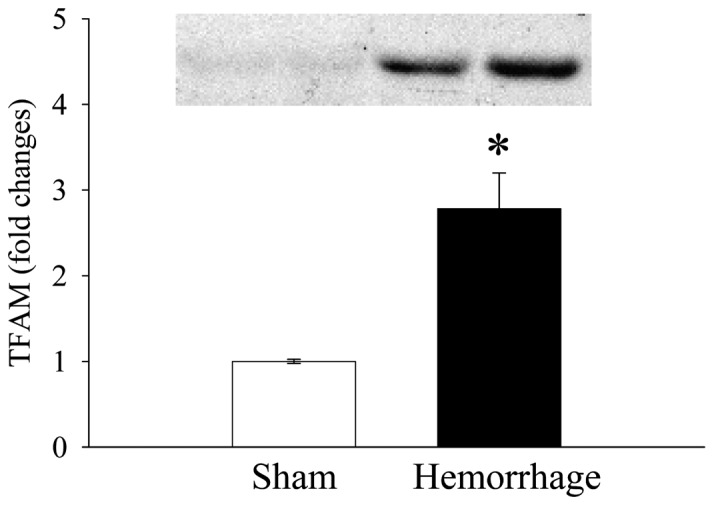
Alterations in serum levels of TFAM in sham-operated animals or hemorrhage animals at 4 h after the completion of resuscitation. Data are presented as means ± SE (n=4 or 6/group) and compared by Student’s t-test: ^*^P<0.05 vs. the sham group. Representative gels of 2 independent observations are presented.

As shown in Fig. 1, a small amount of TFAM protein was detected in the serum of sham-operated animals. At 4 h after hemorrhage and resuscitation, however, serum levels of TFAM were more than doubled (P<0.05, Fig. 1).

### TFAM stimulates TNF-α release in macrophages

**Figure 2:**
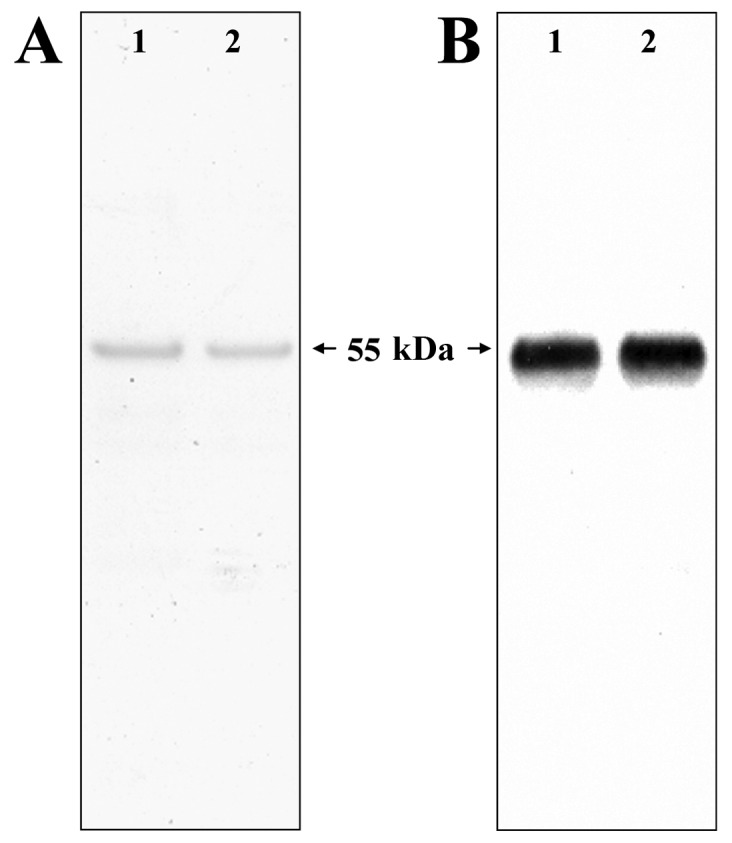
(A) SDS-PAGE analysis of the expressed and purified rat GST-TFAM. Lane 1, purified GST-TFAM (3 μg); lane 2, purified GST-TFAM (1.5 μg); (B) Western blot analysis of the expressed and purified GST-TFAM. The specific antibody recognizes GST-TFAM by western blot analysis. Lane 1, purified GST-TFAM (5 μg from 1st batch); lane 2, purified GST-TFAM (5 μg from 2nd batch).

**Figure 3:**
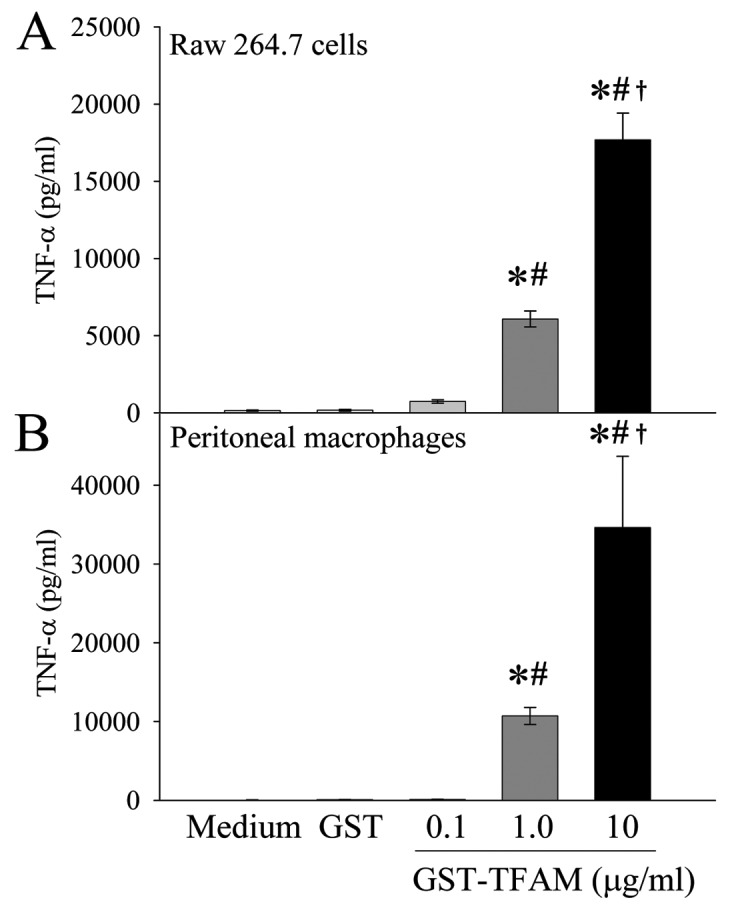
(A) Dose-dependent effects of GST-TFAM on TNF-α secretion in RAW 264.7 cells or (B) peritoneal macrophages isolated from healthy rats. Cells were cultured in DMEM containing various concentrations of GST-TFAM (0.1, 1 and 10 μg/ml) or GST (5 μg/ml) for a period of 4 h. The supernatant levels of TNF-α were measured by ELISA. Data are presented as means ± SE (n=4) and compared by one-way ANOVA and the Student Newman-Keuls test: ^*^P<0.05 vs. medium alone; ^#^P<0.05 vs. GST alone; ^†^P<0.05 vs. 0.1 μg/ml GST-TFAM group.

Triggering the systemic inflammatory response after hemorrhagic shock has been attributed to multiple organ dysfunction (6). We investigated whether the released TFAM induced inflammation. We first expressed and purified recombinant TFAM with a GST-tag (GST-TFAM) in an *E. coli* expression system. The TFAM part in the GST-TFAM fusion protein contains 244 amino acids with a molecular weight of 28.2 kDa. The total calculated size of the GST-TFAM fusion protein is 55.3 kDa. As shown in Fig. 2A, the SDS-PAGE analysis of the fusion protein indicated a single band at ~55 kDa. The purity of our recombinant GST-TFAM was over 99% according to SDS-PAGE method (Fig. 2A). The endotoxin level in the recombinant protein sample was not detectable as measured by Limulus Amebocyte Lysate method (data not shown). Western blot analysis showed that purified GST-TFAM was immunoreactive for specific anti-TFAM antibodies (Fig. 2B). We then stimulated macrophages with recombinant GST-TFAM. As shown in Fig. 3A, addition of GST-TFAM to RAW 264.7 cells, a murine macrophage cell line, caused an increase of TNF-α release in a dose-dependent manner. Since the GST-TFAM fusion protein contains approximately 50% GST, 5 μg/ml GST alone (i.e., 50% of the highest dose of GST-TFAM) was used as a control. As shown in Fig. 3A, the GST control alone had no detectible effect on TNF-α release from RAW 264.7 cells. To confirm this result, we isolated peritoneal macrophages from normal rats. Similarly, GST-TFAM, not GST, dose-dependently increased TNF-α release from primary peritoneal macrophages (Fig. 3B).

### TFAM induces systemic inflammation in rats

**Figure 4:**
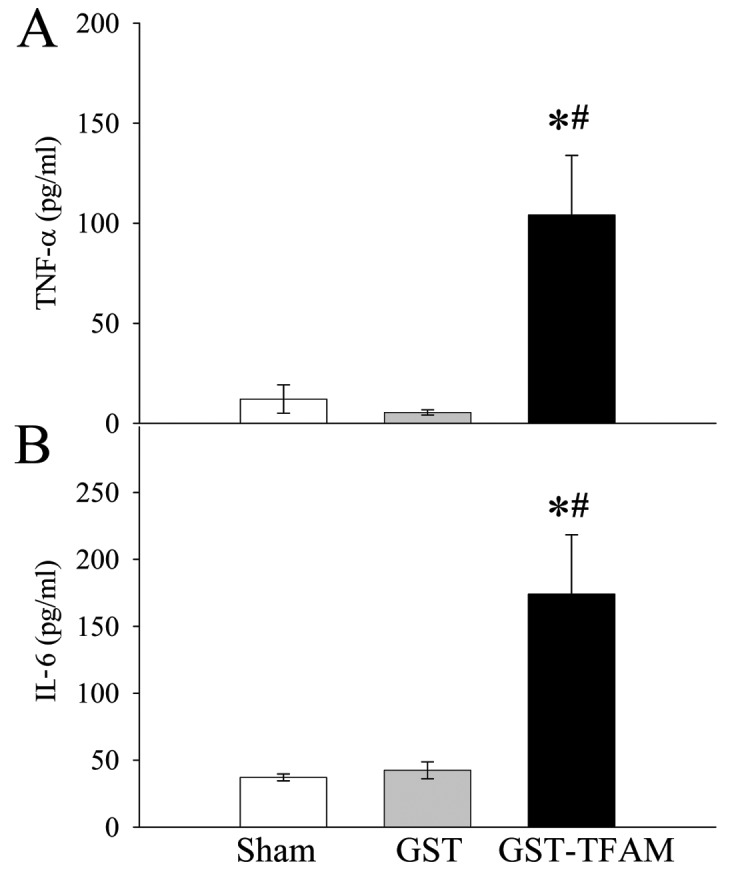
Administration of GST-TFAM increases serum levels of (A) TNF-α and (B) IL-6 in rats. Healthy rats were intravenously administered with GST-TFAM (1 mg/kg BW), GST (0.5 mg/kg BW) or sham (1 ml normal saline). After 5 h, blood samples were collected for measuring serum TNF-α and IL-6 by ELISA. Data are presented as means ± SE (n=3–4) and compared by one-way ANOVA and the Student Newman-Keuls test. ^*^P<0.05 vs. the sham group; ^#^P<0.05 vs. the GST alone group.

**Figure 5:**
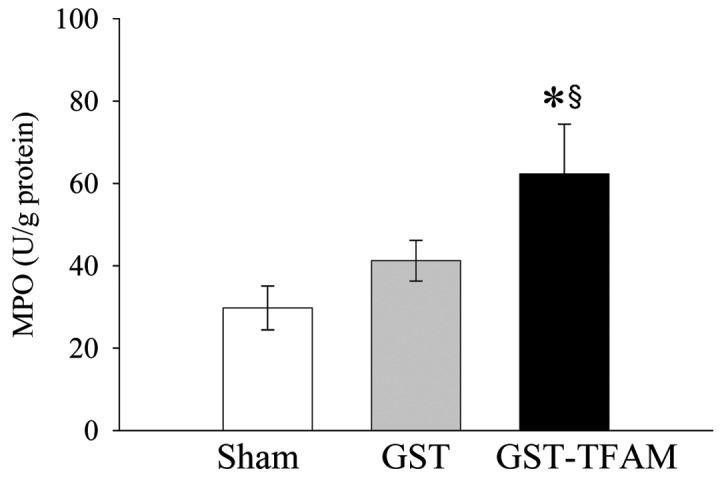
Administration of GST-TFAM increases pulmonary MPO activities in rats. Healthy rats were intravenously administered with GST-TFAM (1 mg/kg BW), GST (0.5 mg/kg BW) or sham (1 ml normal saline). After 24 h, lung samples were collected for measuring MPO activities. Data are presented as means ± SE (n=5–6) and compared by one-way ANOVA and the Student Newman-Keuls test: ^*^P<0.05 vs. the sham group; ^§^P=0.075 vs. the GST alone group.

**Figure 6:**
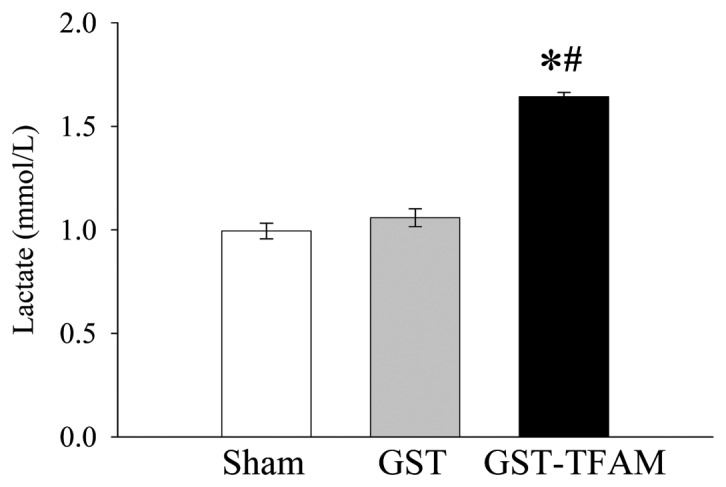
Administration of GST-TFAM increases serum levels of lactate in rats. Healthy rats were intravenously administered with GST-TFAM (1 mg/kg BW), GST (0.5 mg/kg BW) or sham (1 ml normal saline). After 24 h, blood samples were collected for measuring serum lactate. Data are presented as means ± SE (n=4–6) and compared by one-way ANOVA and the Student Newman-Keuls test: ^*^P<0.05 vs. Sham group; ^#^P<0.05 vs. GST alone group.

To determine the effect of TFAM *in vivo*, we administered GST-TFAM to healthy rats. As shown in Fig. 4A, administration of GST-TFAM resulted in a significant increase of serum TNF-α 5 h later. Administration of the same amount of GST, on the other hand, had no effect on serum levels of TNF-α. Similarly, serum levels of IL-6 were also significantly increased by the administration of GST-TFAM, not GST (Fig. 4B). Neutrophil infiltration leads to organ injury after hemorrhagic shock. To determine the effect of TFAM on neutrophil infiltration, we measured the MPO activity in the lungs after its administration. As shown in Fig. 5, a slight but no significant increase in pulmonary MPO activities were observed in GST-treated animals. However, administration of GST-TFAM increased pulmonary MPO activities by 109% (P<0.05, Fig. 5). Circulating levels of lactate, an indicator of systemic hypoxia, often increase after hemorrhagic shock. As shown in Fig. 6, administration of GST-TFAM led to a 65% increase in serum levels of lactate (P<0.05). Administration of GST alone, on the other hand, had no effect on the serum levels of lactate (Fig. 6).

## Discussion

The maintenance of integrity of mitochondrial DNA is important for keeping proper cellular functions both under physiological and pathological conditions. TFAM is a nuclear-encoded transcription factor for mitochondrial DNA. Intracellular TFAM is essential for the maintenance of mitochondrial DNA. It stabilizes mitochondrial DNA through formation of a nucleoid and regulates (or titrates) the amount of mitochondrial DNA. Overexpression of human TFAM in mice increases the amount of mitochondrial DNA and dramatically ameliorates the cardiac dysfunctions caused by myocardial infarction (21). In contrast, our current study provides direct evidence that extracellular TFAM can act as a DAMP and trigger inflammation.

After tissue injury or trauma related to hypoxia, ischemia, or mechanical stress endogenous molecules released from necrotic or apoptotic cells can activate the immune system in a fashion analogous to PAMPs. These molecules are called DAMPs. These endogenous or self-molecules typically function in normal cell homeostasis but are also recognized as danger signals when released into the extracellular space exposing hydrophobic portions of the molecules that are normally hidden in healthy living cells (22,23). To prove TFAM is a DAMP, we first determined whether TFAM can be released into the circulation after hemorrhagic shock. Our result has clearly demonstrated that TFAM, a mitochondrial protein, is upregulated and can be released into the circulation after hemorrhagic shock.

Macrophages play an important role in initiating immune responses (24). Depletion of macrophages by liposomal clodronate prevents LPS-induced cytokine production and subsequent liver damage in the rat (25). In this regard, we stimulated RAW 264.7 cells, a murine macrophage cell line, with recombinant rat GST-TFAM. Our result showed that GST-TFAM dose-dependently increased TNF-α release in RAW 264.7 cells. To further confirm this finding, we isolated primary peritoneal macrophages from healthy rats. Consistent with the findings in RAW 264.7 cells, our result showed that GST-TFAM dose-dependently increased TNF-α release in primary peritoneal macrophages. Thus, extracellular TFAM can stimulate pro-inflammatory cytokine release in cultured macrophages.

Although stimulation of TNF-α release from macrophages by recombinant rat GST-TFAM provided direct evidence of the effect of extracellular TFAM on activating immune cells, these results need to be confirmed *in vivo*. In this regard, we injected recombinant rat GST-TFAM to healthy rats. Our results demonstrated that intravenous injection of GST-TFAM upregulated circulating levels of pro-inflammatory cytokines, increased neutrophil infiltration to the lungs and caused organ injury in healthy animals. Although further studies are needed, this novel discovery may open a whole new area of trauma research. Future studies should be focused on how TFAM is released into the circulation after hemorrhagic shock, the detailed signaling pathway for the pro-inflammatory effects of extracellular TFAM, as well as whether blockade of the TFAM pathway could inhibit inflammatory responses and mitigate organ injury after hemorrhagic shock.

In summary, TFAM was released into the circulation in rats after hemorrhagic shock. Extracellular TFAM stimulated pro-inflammatory cytokine release in cultured RAW 264.7 cells and primary peritoneal macrophages. Intravenously administration of TFAM provoked inflammatory responses and led to organ injury in healthy animals. Thus, TFAM can act as a DAMP and may contribute to the initiation of inflammatory responses after hemorrhagic shock.
